# Energy Requirements of Adult Dogs: A Meta-Analysis

**DOI:** 10.1371/journal.pone.0109681

**Published:** 2014-10-14

**Authors:** Emma N. Bermingham, David G. Thomas, Nicholas J. Cave, Penelope J. Morris, Richard F. Butterwick, Alexander J. German

**Affiliations:** 1 Food Nutrition & Health Team, Food & Bio-based Products, AgResearch Grasslands, Palmerston North, New Zealand; 2 Centre of Feline Nutrition, Massey University, Palmerston North, New Zealand; 3 Institute of Veterinary Animal Biomedical Sciences, Massey University, Palmerston North, New Zealand; 4 WALTHAM Centre of Pet Nutrition, Mars Petcare, Waltham on the Wolds, Leicestershire, United Kingdom; 5 Department of Obesity and Endocrinology, University of Liverpool, Neston, Cheshire, United Kingdom; INIA, Spain

## Abstract

A meta-analysis was conducted to determine the maintenance energy requirements of adult dogs. Suitable publications were first identified, and then used to generate relationships amongst energy requirements, husbandry, activity level, methodology, sex, neuter status, dog size, and age in healthy adult dogs. Allometric equations for maintenance energy requirements were determined using log-log linear regression. So that the resulting equations could readily be compared with equations reported by the National Research Council, maintenance energy requirements in the current study were determined in kcal/kg^0.75^ body weight (BW). Ultimately, the data of 70 treatment groups from 29 publications were used, and mean (± standard deviation) maintenance energy requirements were 142.8±55.3 kcal.kgBW^−0.75^.day^−1^. The corresponding allometric equation was 81.5 kcal.kgBW^−0.93^.day^−1^ (adjusted R^2^ = 0.64; 70 treatment groups). Type of husbandry had a significant effect on maintenance energy requirements (*P*<0.001): requirements were greatest in racing dogs, followed by working dogs and hunting dogs, whilst the energy requirements of pet dogs and kennel dogs were least. Maintenance energy requirements were less in neutered compared with sexually intact dogs (*P*<0.001), but there was no effect of sex. Further, reported activity level tended to effect the maintenance energy requirement of the dog (*P* = 0.09). This review suggests that estimating maintenance energy requirements based on BW alone may not be accurate, but that predictions that factor in husbandry, neuter status and, possibly, activity level might be superior. Additionally, more information on the nutrient requirements of older dogs, and those at the extremes of body size (i.e. giant and toy breeds) is needed.

## Introduction

In 2006, the National Research Council (NRC) published the latest version of the Nutrient Requirements of Dogs and Cats, in which there was considerable detail on maintenance energy requirements of dogs [1]. Based upon a review of the available literature at the time, maintenance energy requirements for adult pet dogs varied between 95 and 200 kcal/kg^0.75^ depending on breed, activity level or husbandry type (i.e., laboratory or home) [1]. Since then, further studies have been published concerning energy requirements of dogs [2], [3], [4]. Determining the energy requirements of pet dogs is a particular challenge, since data from other populations, particularly those from dogs in kennelled environments, are not representative [1]. As a consequence, a number of recent studies have specifically estimated maintenance energy requirements in populations of pet dogs [3], [5], [6], [7], [8].

All studies, both recent and historical, have their place and can provide a valuable contribution. However, limitations were also identified, such as the husbandry of the animals in the study (e.g., colony dogs vs. pet dogs), the signalment of the dogs (i.e., the breed size, age range, sex, and neuter status), activity levels, and the method of measuring energy requirement. When studies are taken in isolation, these limitations can introduce experimental bias, and can affect the way in which the results are interpreted. Meta-analysis is an advanced statistical procedure whereby the results of multiple experiments are combined in order to minimise the effects of bias in the individual studies [9]. The resultant dataset is larger, making the findings more robust, and differences in experimental conditions amongst individual studies can be offset, increasing the accuracy of extrapolation to the wider population. Meta-analyses are now commonplace in medical science, and have rapidly become an important tool to help clinicians to determine the best clinical information, to assist healthcare policy makers when assessing the risks and benefits of interventions, to help funders in deciding whether new research in a particular field is warranted, and to assist the editors of scientific journals to determine if there is a need to publish a new manuscript in a particular field [10]. Although most commonly used to determine the efficacy of therapeutic interventions, the principles can readily be used to assess other scientific questions on which sufficient published data exist.

Recently, a meta-analysis was conducted examining maintenance energy requirements of cats [11]. Its key finding was that the maintenance energy requirements reported by the NRC [1] over-estimate the true energy requirements of the domestic cat. It also highlighted that maintenance energy requirements are inaccurately predicted if based upon body weight (BW) alone, with age, sex and neuter status of the cat also affecting requirements [11]. However, to the authors' knowledge such an approach has not previously been undertaken to examine maintenance energy requirements of dogs. Therefore, the objective of the present review was to conduct a meta-analysis of the energy requirements for maintaining BW in adult domestic dogs, and specifically pet dogs, in order to (a) determine predicted differences in energy requirements with BW, and (b) determine the factors that influence the requirements. For ease of comparison with the NRC [1], we have reported energy requirements in kcal/kg BW, rather than using System Internationale units (MJ/kg BW).

## Materials and Methods

### Study protocol

This study was a meta-analysis of publications concerning metabolisable energy requirements for maintenance (also known as: maintenance energy requirements) in adult dogs, and has been reported in accordance with the Preferred Reporting Items for Systematic reviews and Meta-Analyses (PRISMA) statement [12] with reference to the explanation and elaboration document [10]. Two of the authors (EB, AG), met in November 2012 to conceptualise the study and agree in advance on protocol. This information was subsequently shared with and agreed by all authors before proceeding. Although the protocol of the current study was not made publically available prior to the study, the approach was similar to a meta-analysis published recently on energy requirements of domesticated cats [11].

### Information sources and searches

The primary author (EB) searched the scientific literature electronically in order to identify publications that estimated the maintenance energy requirements of healthy, adult, domesticated dogs. Online resources searched included OVID databases (Medline, BIOSIS, FSTA, CAB Abstracts), SCOPUS, and PUBMED. The search terms used to identify suitable publications are listed in Table 1, and included relevant terms covering dog, energy, maintenance energy requirements, obesity, and weight loss and gain. The electronic searches commenced on 13/11/2012, and the last search was performed on 26/11/2012.

**Table 1:** Search terms.

Term	Sub-terms
Dog*	Pet, working, police, farm, colony, laboratory
Energy*	Requirements, expenditure, intake
Maintenance energy requirements	
Obesity	
Weight loss or gain	

*Denotes ‘wild card’ search.

### Eligibility criteria

A ‘publication’ was defined as a distinct piece of published work, be it full paper or research communication (abstract) at a scientific meeting. The term ‘treatment group’ was defined as a distinct group of dogs within a publication. Groups of dogs within a publication could include those with differing baseline characteristics (e.g., breed), husbandry type (e.g., racing [including sight hounds and arctic breeds], working, hunting, kennel, or pet), activity (e.g., resting, low, moderate, or high), or method used to determine maintenance energy requirements. The authors of each publication defined the treatment groups. Only publications in the English language were considered, but no date limits were set for inclusion. To maximise the number of publications available, original prospective studies using a variety of study types were allowed, including prospective cohort studies, observational studies, and case-control studies. However, maintenance energy requirements based on survey data (e.g., information gathered by questionnaire) were not included, in view of concerns with the reliability of such data [13], [14], [15]. In addition, various experimental designs were allowed, including single group, parallel group, crossover, and Latin square designs. Further, various methods for estimation of metabolisable energy were allowed including feeding experiments (FE), indirect calorimetry (IC), and tracer studies including doubly-labelled water (DLW). Whilst methodologies, such as IC and DLW, estimate of energy expenditure rather than energy requirements *per se*, for the purpose of this meta-analysis, it was assumed that these estimates approximated one another. In the case of FE studies, in order to be certain that the reported energy requirements truly represented maintenance energy requirements, only studies of greater than 7 days duration, with a maximum allowed variation in BW of ±5%, were included [16]. Given that pathological conditions might influence maintenance energy requirements, publications were only included if dogs were free from disease, and were not overweight (e.g., BCS<6/9 [BCS<4/5]). Finally, publications where the primary focus of the research was on the method of estimation itself (e.g., method validation studies) were not included, since this focus might have had an undue influence on the results generated. For most groups, activity level was subjectively classed as low (<1h/day), medium (1–3 h/day), high (>3 h/day) based upon previously defined criteria [17]. However, activity levels were classed as ‘resting’ when activity was deemed to be negligible, based upon the fact that measurements were made either when lying still in lateral recumbency, or when cage rested.

### Study selection and data collection process

The primary author (ENB) reviewed all publications identified from the electronic search, and assessed study eligibility in a standardised, unblinded, manner. A copy of all eligible publications was first obtained, either as a portable document format (PDF) file, or as a photocopy of the original paper document. If the primary author could not access the material, a second author, who worked at a different academic institution (AJG), then attempted to access it. If neither of the authors could access the publication, the corresponding author was contacted, and a copy requested. A decision was made to contact each corresponding author twice, and the publication was deemed to be unavailable if there was either no response or the request was refused.

The primary author extracted relevant data from all eligible publications that were available. This included dog-specific information, environmental information, and information required for maintenance energy requirement calculations (Table 2). Dog size (breed size; toy, small, medium, large, giant; determined by breed of the dog) was allocated according to the criteria described previously [18], [19]. Biological age was defined as a function of chronological age and breed, since smaller dogs typically have longer life expectancy compared with giant breeds [19], [20]. Given that body condition score was only reported in 4 publications [4], [21], [22], [23], and was never abnormal (2/9<BCS<6/9 [1/5<BCS<4/5]), this information was not included in the final dataset. Data were entered into a computer spreadsheet (Excel version 2010, Microsoft, Redmond, USA). The daily maintenance energy requirements of all publications were plotted against bodyweight in order to assess any outliers in the data set; a decision was made, based on eligibility criteria, whether or not to include the publications and their treatment groups, and outliers were removed from the dataset at this point (Table 2). All decisions regarding inclusion of each publication and treatment group were subsequently reviewed and verified by a second author (AJG). Any discrepancies, errors or omissions were resolved by consensus between the two authors.

**Table 2:** Literature used to determine the maintenance energy requirements (MER; kcal.day^−1^) for adult dogs.

Reference	Dogs (n)	Study Length (d)	Breed	Breed Size^1^	Mean Age (years)	Age-range	Sex	Neuter Status	Husbandry	Activity level^2^	Method	Mean BW (kg)	SD	MER (kcal.day^−1^)	SD	Used in dataset
Ahlstrom et al [63]	1	7	Brittany Spaniel	Medium	2.5	Young adult	F	Mix	Hunting	High	DLW	16.0		1092.0		Yes
	1	7	Brittany Spaniel	Medium	5.5	Young adult	F	Mix	Hunting	High	DLW	14.4		2183.6		Yes
	1	7	Brittany Spaniel	Medium	2.5	Young adult	F	Mix	Hunting	High	DLW	15.2		899.1		Yes
	1	7	Brittany Spaniel	Medium	5.5	Young adult	F	Mix	Hunting	High	DLW	14.1		1416.1		Yes
	1	7	Brittany Spaniel	Medium	2.5	Young adult	F	Mix	Hunting	High	DLW	15.6		1017.7		Yes
	1	7	Brittany Spaniel	Medium	5.5	Young adult	F	Mix	Hunting	High	DLW	16.8		1129.5		Yes
Ahlstrom et al [64]	8	3	Hunting	Not known		Not given	M+F	Mix	Hunting	High	DLW	19.8	4.7	2620.8	268.8	Yes
Anantharaman-Barr [48]	6	30	Mixed	Medium		Not given	F	Mix	Kennel	Low	IC	10.9		405.0		Yes
	6	30	Mixed	Medium		Not given	F	Mix	Kennel	Low	IC	11.4		338.0		Yes
	6	30	Mixed	Medium		Not given	F	Mix	Kennel	Low	IC	11.7		412.0		Yes
Ballevre et al. [65]	2	11	Beagle	Medium	8.0	Adult	F	I	Kennel	Low	DLW	14.0		789.7		Yes
Blaza [66]	5	21	Mixed	Not known		Not given	M+F	Mix	Kennel	Low	FE	26.0		1340.0		Yes
	5	21	Mixed	Not known		Not given	M+F	Mix	Kennel	Low	FE	26.0		1461.0		Yes
Bouthegourd et al [67]	5	7	Labrador Retriever	Large		Not given	M+F	Mix	Kennel	Low	Allometric			*		No; no BW reported
	7	7	Great Dane	Giant		Not given	M+F	Mix	Kennel	Low	Allometric			*		No; no BW reported
	9	7	German Shepherd	Large		Not given	M+F	Mix	Kennel	Low	Allometric			*		No; no BW reported
Butterwick & Hawthorne [17]	7	56	Border collie	Medium		Not given	M+F	Mix	Pet	Low	FE	18.0	2.9	1048.5	257.7	Yes: BW supplied by author.
	28	56	Border collie	Medium		Not given	M+F	Mix	Pet	Moderate	FE	18.7	4.0	971.5	229.3	Yes: BW supplied by author.
	5	56	Border collie	Medium		Not given	M+F	Mix	Pet	High	FE	23.3	4.2	1878.2	698.8	Yes: BW supplied by author.
	40	56	Border collie	Medium		Not given	M+F	Mix	Pet	Low	FE	19.2	4.1	1087.3	361.3	Yes: BW supplied by author
	11	56	Border collie	Medium		Not given	M+F	Mix	Working	Low	FE	18.0	2.9	1249.0	280.5	Yes: BW supplied by author
	10	56	Border collie	Medium		Not given	M+F	Mix	Working	Moderate	FE	16.9	3.5	1534.0	165.9	Yes: BW supplied by author
	7	56	Border collie	Medium		Not given	M+F	Mix	Working	High	FE	19.1	1.2	1589.7	540.0	Yes: BW supplied by author
	28	56	Border collie	Medium		Not given	M+F	Mix	Working	Low	FE	17.8	2.9	1431.2	351.9	Yes: BW supplied by author
Decombaz et al. [45]	13	9	Husky	Large		Not given	M+F	Mix	Racing	High	DLW	19.0	1.8	4014.0	1542.0	Yes
	13	9	Husky	Large		Not given	M+F	Mix	Racing	High	FE	19.0	1.8	2089.0	219.0	Yes
Finke [68]	6	378	Beagle	Medium	4.1	Young adult	M+F	Mix	Kennel	Low	FE	13.2	2.3	816.0	154.5	Yes
	6	378	Husky	Large	9.2	Old	M+F	Mix	Kennel	Low	FE	20.9	3.9	1341.1	210.7	Yes
	6	378	Labrador	Large	3.2	Young adult	M+F	Mix	Kennel	Low	FE	31.4	4.4	1200.0	137.0	Yes
Finke [69]	6	420	Beagle	Medium	1.6	Young adult	F	Mix	Kennel	Low	FE	10.7	1.4	843.4	28.4	Yes
	8	420	Beagle	Medium	6.6	Adult	F	Mix	Kennel	Low	FE	10.8	1.7	673.8	61.0	Yes
	5	420	Beagle	Medium	12.4	Old	F	Mix	Kennel	Low	FE	11.7	1.4	689.8	70.8	Yes
Frankel & Bell [70]	1	8	Kelpie Sheepdog	Medium		Not given	M	Mix	Pet	Low	DLW	17.5		1425.7		Yes
Hill et al. [44]	4	112	Greyhound	Large	1.8	Young adult	M+F	Mix	Racing	Low	FE	33.9	2.4	2177.6	140.5	Yes
	4	112	Greyhound	Large	1.8	Young adult	M+F	Mix	Racing	Low	FE	33.9	1.9	2163.6	154.5	Yes
Hill et al. [71]	4	154	Greyhound	Large	3.5	Young adult	M+F	Mix	Racing	Low	FE	32.2	2.9	2027.6	392.0	Yes
	4	154	Greyhound	Large	3.5	Young adult	M+F	Mix	Racing	Low	FE	32.8	2.5	2110.7	233.0	Yes
Hinchcliff et al. [25]	8	3	Husky	Large	3.0	Young adult	M+F	I	Racing	High	DLW	24.3	2.5	11257.2	1410.1	No: outlier
	4	3	Husky	Large	3.0	Young adult	M+F	I	Racing	Low	DLW	22.8	2.8	2509.6	812.6	Yes
Jeusette et al. [21]	4	42	Beagle	Medium	2.0	Young adult	F	I	Kennel	Low	FE	14.0	0.3	1245.5	22.5	Yes
	4	182	Beagle	Medium	2.0	Young adult	F	N	Kennel	Low	FE	13.7	0.1	861.2	13.6	Yes
	4	112	Beagle	Medium	2.0	Young adult	F	N	Kennel	Low	FE	13.7	0.1	*	*	No; BW not stable
Jeusette et al. a [22]	12	30	Beagle	Medium	4.4	Young adult	M+F	Mix	Kennel	Low	FE	12.7	0.7	787.9	0.0	Yes
	12	30	Beagle	Medium	4.7	Young adult	M+F	Mix	Kennel	Low	FE	21.9	0.8	*	*	No: Obese
	12	30	Beagle	Medium	4.7	Young adult	M+F	Mix	Kennel	Low	FE	21.5	0.8	*	*	No; Obese
Jeusette et al. b [23]	12	30	Beagle	Medium	4.4	Young adult	M+F	Mix	Kennel	Low	FE	12.7	0.7	787.9	0.0	Yes;
	12	30	Beagle	Medium	4.7	Young adult	M+F	Mix	Kennel	Low	FE	21.9	0.8	*	*	No: obese
	12	30	Beagle	Medium	4.7	Young adult	M+F	Mix	Kennel	Low	FE	21.5	0.8	*	*	No: obese
	12	30	Beagle	Medium	4.7	Young adult	M+F	Mix	Kennel	Low	FE	14.8	0.3	*	*	No: obese;
	12	180	Beagle	Medium	4.7	Young adult	M+F	Mix	Kennel	Low	FE	14.8	0.3	*	*	No: obese
Jeusette et al. c [72]	4	42	Beagle	Medium	2.0	Young adult	F	I	Kennel	Low	FE	14.0	0.3	1396.0	22.5	Yes
	4	180	Beagle	Medium	2.0	Young adult	F	N	Kennel	Low	FE	13.7	0.1	975.2	22.1	Yes
Jewell et al. [73]	3	180	Beagle	Medium		Not given	F	I	Kennel	Low	FE	15.0		*	*	No; obese
	3	180	Beagle	Medium		Not given	F	I	Kennel	Low	FE	15.0		*	*	No; obese
	3	180	Beagle	Medium		Not given	F	I	Kennel	Low	FE	15.0		*	*	No; obese
	3	180	Beagle	Medium		Not given	F	I	Kennel	Low	FE	15.0		*	*	No; obese
Kendall et al [27]	6	182	Beagle	Medium		Not given	M	I	Kennel	Low	FE	14.5		1041.1	9.135	Yes
Kienzle & Rainbird [74]	26	35	Newfoundland	Giant	5.0	Adult	M+F	I	Kennel	Low	FE			*		No; no BW reported
	8	35	Great Dane	Giant	1.0	Young adult	M+F	I	Kennel	Low	FE			*		No; no BW reported
	3	35	Great Dane	Giant	5.0	Adult	M+F	I	Kennel	Low	FE			*		No; no BW reported
	15	35	Labrador	Large	1.0	Young adult	M+F	I	Kennel	Low	FE			*		No; no BW reported
	13	35	Labrador	Large	2.0	Young adult	M+F	I	Kennel	Low	FE			*		No; no BW reported
	45	35	Labrador	Large	5.0	Adult	M+F	I	Kennel	Low	FE			*		No; no BW reported
	14	35	Labrador	Large	7.0	Adult	M+F	I	Kennel	Low	FE			*		No; no BW reported
	4	35	Briard	Large	1.0	Young adult	M+F	I	Kennel	Low	FE			*		No; no BW reported
	9	35	Briard	Large	5.0	Adult	M+F	I	Kennel	Low	FE			*		No; no BW reported
	7	35	Beagle	Medium	2.0	Young adult	M+F	I	Kennel	Low	FE			*		No; no BW reported
	11	35	Beagle	Medium	5.0	Young adult	M+F	I	Kennel	Low	FE			*		No; no BW reported
	32	35	Beagle	Medium	7.0	Adult	M+F	I	Kennel	Low	FE			*		No; no BW reported
	9	35	Cairn Terrier	Small	1.0	Young adult	M+F	I	Kennel	Low	FE			*		No; no BW reported
	8	35	Dachshund	Small	1.0	Young adult	M+F	I	Kennel	Low	FE			*		No; no BW reported
Laflamme et al [75]	9	42	Mixed	Not known	2.5	Not given	M+F	Mix	Kennel	Low	FE	21.0		1723.0		Yes;
	9	42	Mixed	Not known	10.9	Not given	M+F	Mix	Kennel	Low	FE	17.0		1082.0		Yes;
Larson et al. [76]	24	2190	Labrador	Large	9.0	Adult	M+F	Mix	Kennel	Low	FE			*		No; no BW reported
Larsson et a l [3]	2	140	English Springer Spaniel	Large	8.5	Adult	M	Mix	Pet	Resting	Tracer	24.0	0.0	1285.4	102.6	Yes
	2	140	English Springer Spaniel	Large	8.5	Adult	M	Mix	Pet	Moderate	Tracer	24.0	0.0	1421.5	5.4	Yes
	2	140	German Short-haired pointer	Large	6.0	Adult	M	Mix	Pet	Resting	Tracer	31.5	2.1	1695.4	92.2	Yes
	2	140	German Short- haired pointer	Large	6.0	Adult	M	Mix	Pet	Moderate	Tracer	31.5	2.1	1695.4	92.2	Yes
	2	140	Beagle	Medium	4.0	Young adult	F	Mix	Pet	Resting	Tracer	11.7	0.1	543.6	9.7	Yes
	2	140	Beagle	Medium	4.0	Young adult	F	Mix	Pet	Moderate	Tracer	11.7	0.1	715.2	34.2	Yes
Ogilvie et al. [77]	30	105	Mixed	Not known	5.4	Not given	M+F	Mix	Pet	Low	IC	28.0		1526.0	436.8	Yes
	30	105	Mixed	Not known	5.4	Not given	M+F	Mix	Pet	Low	IC	28.0		1610.0	417.2	Yes
Ogilvie et al. [78]	30	35	Mixed	Not known	5.4	Not given	M+F	Mix	Pet	Low	IC	28.0		1509.2	448.0	Yes
O'Toole et al. [7]	7	2	Mixed	Not known	5.5	Not given	M+F	Mix	Pet	Resting	IC	27.4		*		No; data in graphs
O'Toole et al. [8]	10	150	Mixed	Not known	4.0	Not given	M+F	N	Pet	Resting	IC	28.1		*		No; data in graphs
	34	150	Mixed	Not known	5.0	Not given	M+F	Mix	Pet	Resting	IC	24.0		*		No; data in graphs
	17	150	Mixed	Not known	4.0	Not given	M+F	Mix	Pet	Resting	IC	30.0		*		No; data in graphs
	16	150	Mixed	Not known	2.0	Not given	M+F	Mix	Pet	Resting	IC	29.0		*		No; data in graphs
Owens [79]		168	Pointer	Large	3.8	Young adult	M	Mix	Kennel	Low	FE			*		No; no BW reported
		168	Pointer	Large	3.8	Young adult	F	Mix	Kennel	Low	FE			*		No; no BW reported
		168	Labrador	Large	3.8	Young adult	M	Mix	Kennel	Low	FE			*		No; no BW reported
		168	Labrador	Large	3.8	Young adult	F	Mix	Kennel	Low	FE			*		No; no BW reported
Patil & Bisby [80]	14	56	Mixed	Not known	5.5	Not given	M+F	Mix	Pet	Low	FE	24.0	10.0	1258.0	335.0	Yes
	22	56	Mixed	Not known	6.5	Not given	M+F	Mix	Kennel	Low	FE	20.0	9.0	1284.0	501.0	Yes
Pouteau et al. [81]	6	11	Beagle	Medium	5.2	Young adult	M+F	Mix	Kennel	Low	DLW	17.9	0.8	1148.1	127.4	Yes
	6	11	Beagle	Medium	5.2	Young adult	M+F	Mix	Kennel	Low	IC	18.3	1.3	801.5	129.5	Yes
Reynolds et al [82]	8	84	Husky	Large	4.0	Young adult	M+F	Mix	Kennel	Low	FE	19.9	1.4	1430.8	157.6	Yes
	8	84	Husky	Large	4.0	Young adult	M+F	Mix	Kennel	Low	FE	20.6	1.7	1497.6	460.3	Yes
	8	84	Husky	Large	4.0	Young adult	M+F	Mix	Kennel	High	FE	18.9	1.3	1270.1	491.8	Yes
	8	84	Husky	Large	4.0	Young adult	M+F	Mix	Kennel	High	FE	19.6	1.3	1452.4	576.5	Yes
	8	84	Husky	Large	4.0	Young adult	M+F	Mix	Kennel	High	FE	19.3	1.0	1279.6	185.6	Yes
	8	84	Husky	Large	4.0	Young adult	M+F	Mix	Kennel	High	FE	20.7	1.7	1471.8	199.1	Yes
Serisier et al. [4]	42	196	Miniature	Not known	4.0	Young adult	F	Mix	Kennel	Low	FE	5.0		375.6		Yes
Speakman et al. [83]	35	2920	Papillon	Toy	7.2	Adult	M+F	Mix	Kennel	Resting	DLW	3.0	0.5	206.0	17.7	Yes
	35	2920	Labrador	Large	8.2	Adult	M+F	Mix	Kennel	Resting	DLW	29.8	4.2	1385.5	164.7	Yes
	35	2920	Great Dane	Giant	5.8	Adult	M+F	Mix	Kennel	Resting	DLW	62.8	8.7	3020.3	256.0	Yes
Sunvold et al. [5]	38	70	Mixed	Not known		Not given	M+F	Mix	Pet	Low	FE	60.7	4.8	897.6	51.4	No; survey
	48	70	Mixed	Not known		Not given	M+F	Mix	Pet	Low	FE	51.4	3.9	855.0	45.2	No; survey
	30	70	Mixed	Not known		Not given	M+F	Mix	Pet	Low	FE	50.4	5.4	809.4	61.8	No; survey
	57	70	Mixed	Not known		Not given	M+F	Mix	Pet	Low	FE	40.0	4.1	860.9	52.6	No; survey
Taylor et al [58]	12	112	Mixed	Not known	>8	Not given	M+F	Neu	Kennel	Low	FE			*		No; no BW reported
	12	112	Mixed	Not known	<6	Not given	M+F	Neu	Kennel	Low	FE			*		No; no BW reported
	5	112	Cairn Terrier	Small	>8	Adult	M+F	Neu	Kennel	Low	FE			*		No; no BW reported
	5	112	Cairn Terrier	Small	<6	Young adult	M+F	Neu	Kennel	Low	FE			*		No; no BW reported
	3	112	Labrador Retriever	Large	>8	Adult	M+F	Neu	Kennel	Low	FE			*		No; no BW reported
	3	112	Labrador Retriever	Large	<6	Adult	M+F	Neu	Kennel	Low	FE			*		No; no BW reported
	2	112	Beagle	Medium	>8	Adult	M	Neu	Kennel	Low	FE			*		No; no BW reported
	2	112	Beagle	Medium	<6	Young adult	F	Neu	Kennel	Low	FE			*		No; no BW reported
	2	112	Mixed	Small	>8	Adult	M+F	Neu	Kennel	Low	FE			*		No; no BW reported
	2	112	Mixed	Small	<6	Young adult	M+F	Neu	Kennel	Low	FE			*		No; no BW reported
Thes et al [6]	61		Mixed	Not known	4.0	Not given	M+F	Mix	Pet	Low	FE			*		No; survey data; no BW reported
Walters [26]	20	0.3	Mixed	Not known	6.3	Not given	M+F	Mix	Pet	Low	IC	25.9		1068.6	275.3	Yes
Yoo et al. [84]	10	30	Labrador	Large	2.2	Young adult	M	I	Kennel	Resting	IC	32.0	3.6	1770.5	876.6	Yes
	10	30	Labrador	Large	2.2	Young adult	M	I	Kennel	Resting	IC	31.6	3.6	1957.8	1226.9	Yes

1 Size subjectively scored as toy, small, medium, large, and giant, using the criteria of Hawthorne et al 2004 [18] and Hosgood & Scholl 1998 [85].

2 For most groups, activity level was classed as low (<1 h/day), medium (1–3 h/day), high (>3 h/day) based upon the criteria of Butterwick and Hawthorne (1998) [7]; however, activity levels were classed as ‘resting’ when measurements were taken either when lying still in lateral recumbency or when cage rested. BCS: body condition score; BW: body weight; DLW: doubly-labelled water; F: female; FE: feeding experiment; I: intact; IC: indirect calorimetry; M: male; MER: maintenance energy requirement; Mix: study contains a mix of dogs with different neuter status; Mixed: either dogs of mixed breeding or a range of breeds represented; Neu: neutered; SD: standard deviation.

### Data handling and statistical analysis

The primary author conducted all statistical analyses using computer software (Microsoft Excel 2010, and GenStat 15^th^ Edition SP1). All data are reported as mean (standard deviation, SD or standard error, SE) or median (range), as appropriate. Data were tested for normality, by assessing residual plots of the data, and were found to be of normal distribution. The level of statistical significance was set at *P*<0.05 for two-sided analyses.

In order to account for any inter-publication variability a weighted mixed model analysis using restricted maximum likelihood (REML; GenStat Version 15) was used, where the weights for each observation were inversely proportional to the stated standard deviation (1/SD) quoted in the publication. An average standard deviation of all the data was used if the standard deviation (or alternate measure of error, e.g., SEM, SE) was not published. The publication was considered to be a random effect in the model, whilst fixed effects included activity level, sex, neuter status, method, age-range, husbandry and size.

The allometric equation Y = aBW^b^ were used to determine the relationship between the amount of metabolisable energy required for maintenance and the bodyweight. In this equation, Y =  metabolisable energy required for maintenance (kcal), BW =  bodyweight (kg), and b =  the allometric exponent [24]. Additionally, a regression model was used to determine the relationship between log BW (log kg) and log maintenance energy requirement (log kcal/d) [24]. In order to report data on a “kcal.kgBW^−1^” basis, the log data generated by Genstat were back-transformed using the inverse of log-base10. Data are, therefore, expressed as an energy equivalent (the coefficient), and the algometric exponent which is used to adjust BW, and the adjusted R^2^ value (a measure of fit of the model). For all equations, a subjective assessment of the suitability was made based upon the adjusted R^2^ value, with values <0.50, 0.50–0.70, and>0.70 representing poor, moderate and reasonable fit for the model, respectively.

## Results

### Study selection and study characteristics

**Figure 1:**
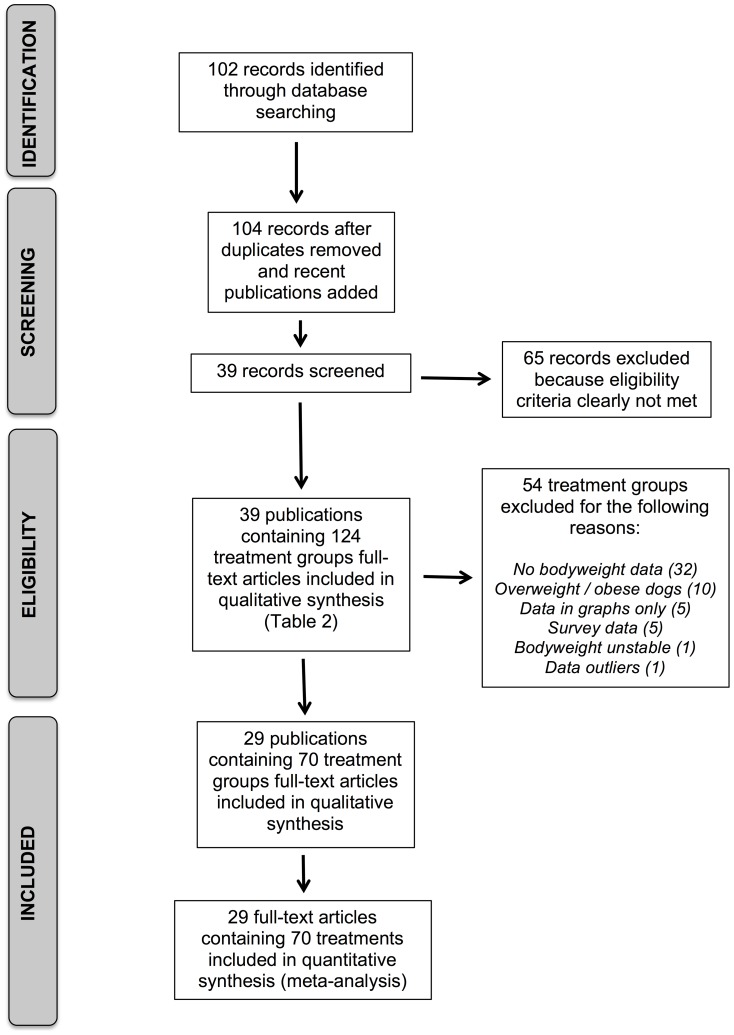
Summary of database searching and inclusion of final groups.

After adjusting for duplicate records, the initial searches identified a total of 102 publications, with an additional two publications identified from interactions at conferences [4], [6] increasing the dataset to 104 publications (Figure 1). Abstracts and titles of these publications were reviewed, and 65 were discarded because they did not meet the eligibility criteria. Therefore, 39 papers that contained appropriate subject matter remained (Table 2). The primary author was able to locate full text versions of all but 13 of these publications, 8 of which were successfully located by a second author (AG). The corresponding authors of the remaining 5 publications were then contacted, by email and, for each one, full-text copies were successfully accessed and included in the analysis. The primary author then screened all 39 publications, in detail, for relevance and 124 treatment groups were identified (Table 2). This was reduced to 29 publications and 70 treatment groups after the removal of 54 treatment groups (Figure 1), most commonly because the publication did not include bodyweight data (32 treatment groups). The remaining treatment groups were removed because dogs were classed as overweight or obese (10 treatment groups), data were reported in graphical form only (5 treatment groups), maintenance energy requirements were based on survey data (5 treatment groups), bodyweight was unstable (1 treatment group), or because the publications were deemed to be outliers (1 treatment group with energy requirements of 11257 kcal/day [25]). Therefore, the final dataset comprised a total of 29 publications, with 70 treatment groups, and comprising a total of 713 dogs (Spreadsheet S1). The median study duration was 56 days (range 0.3 to 2920 days); the study with the shortest duration was an indirect calorimetry study [26]. In this final dataset, energy requirement was determined by FE (39 treatment groups), DLW (15 treatment groups), other tracer studies (6 treatment groups), or by IC (10 treatment groups). For the feeding studies, maintenance energy requirement was determined from the amount of food consumed and the metabolisable energy content of the diet. For this, metabolisable energy content was measured by feeding trials and bomb calorimetry (8 treatment groups) as previously described [27], [28], or calculated from proximate analysis of the diets and use of predictive equations using modified Atwater factors (29 treatment groups). In the remaining two treatment groups, the method by which maintenance energy requirement was determined was not given.

### Dogs

#### Signalment

Age data were reported for 44 treatment groups. Median age was 4.1 years (range 1.6–12.4 years; Table 3). Most treatment groups were classified as young adult dogs (33 treatment groups), with lesser numbers classified as adult (9 treatment groups) and old (2 treatment groups) dogs (Table 3). Twenty-six treatment groups did not specify the age of the dogs studied (Table 3). As indicated in Table 2, the majority of publications either did not report the sex or neuter status of the dogs used, or used a mixed group (42 publications for sex and 61 publications for neuter status). Twenty treatment groups were female only, whilst 8 were male only. For neuter status, 7 treatment groups comprised only intact dogs, whilst 2 treatment groups comprised only neutered dogs. A range of breeds were represented in the various treatment groups including Beagle (16 treatment groups), Border collie (8 treatment groups), Brittany spaniel (6 treatment groups), English springer spaniel (2 treatment groups), German short-haired pointer (2 treatment groups), Great Dane (1 treatment group), Greyhound (4 treatment groups), mixed hunting dogs (1 treatment group; exact breed not reported), Husky (9 treatment groups), Kelpie sheepdog (1 treatment group), Labrador retriever (4 treatment groups), miniature dogs of various breeds (1 treatment group), mixed breed (13 treatment groups, comprising studies with dogs of mixed breeding [10 treatment groups] or those where a range of breeds was used [3 treatment groups]), and Papillon (1 treatment group). Breeds were classed as medium (34 treatment groups), large (22 treatment groups), giant (1 treatment group) and toy (1 treatment group). For the remaining treatment groups, it was not possible to classify their breed size, usually because a mixture of dog breeds was used.

**Table 3:** Maintenance energy requirements (MER) and bodyweights of the dogs in the final dataset.

			MER (kcal.day^−1^)				BW				MER (kcal.kgBW^−1^.day^−1^)				MER (kcal.kgBW^−0.75^.day^−1^)			
Factor	Sub-group	N	Mean	Min	Max	SD	Mean	Min	Max	SD	Mean	Min	Max	SD	Mean	Min	Max	SD
Overall		70	1351	206	4014	639.8	20.2	3	62.8	8.8	68.8	29.7	211.3	26.8	142.8	54.5	441.1	55.3
Size^1^																		
Giant		1	3020	3020	3020	*	62.8	62.8	62.8	*	48.1	48.1	48.1	*	135.4	135.4	135.4	*
Large		22	1784	1200	4014	622	25.9	18.9	33.9	6	72.6	38.2	211.3	35.2	160.8	90.5	441.1	70.8
Medium		34	1036	338	2184	409	15	10.7	23.3	3.1	68.2	29.7	151.8	22.4	134.2	54.5	295.7	45.1
Toy		1	206	206	206	*	3	3.0	3.0	*	68.2	68.2	68.2	*	89.9	89.9	89.9	*
Not known		12	1435	375.6	2621	508	22.4	5.0	28.0	14.4	65.5	41.3	132.6	29.7	138.9	90.1	279.6	58.8
Age range																		
Old		2	1015	690	1341	461	16.3	11.7	20.9	6.5	61.6	59	64.2	3.7	123.1	109	137.2	19.9
Adult		9	1353	206	3020	800	25.7	3.0	62.8	17.1	55.8	46.5	68.2	6.8	117.9	89.9	135.4	14.3
Young adult		33	1314	376	2510	541	19.1	5.0	33.9	7.9	70.8	38.2	151.8	20.8	144.8	85.9	295.7	41.1
Not given		26	1423	338	4014	701	20	10.9	28	11.9	71.5	29.7	211.3	38.8	150.3	54.5	441.1	79.8
Sex																		
F		20	900	338	2184	443	12.9	5.0	16.8	2.6	68.9	29.7	151.8	27.2	130.3	54.5	295.7	54
M		8	1537	1041	1958	297	25.8	14.5	32	7	61.4	53.6	81.7	10.3	136.3	118.6	167	15.1
Mix		42	1530	206	4014	664	25.1	3.0	62.8	11.9	65.6	14.8	211.3	31.5	140.9	41.3	441.1	64.6
Neuter Status																		
I		7	1530	790	2510	590	20.4	14.0	32.0	8.4	77.8	55.3	110.1	22	161.9	109.1	240.5	44.1
Neu		2	918	861	975	81	13.7	13.7	13.7	0	67	62.9	71.2	5.9	128.9	120.9	137	11.3
MIX		61	1344	206	4014	643	20.4	3.0	62.8	11.5	67.8	29.7	211.3	29.5	141	54.5	441.1	59.9
Husbandry																		
Racing		7	2442	2028	4014	711	27.7	19	33.9	7.1	98.1	63	211.3	54.4	217.7	150	441.1	105.8
Working		4	1451	1249	1590	150	18	16.9	19.1	0.9	81	69.4	90.8	8.9	166.5	142.9	184.0	17.5
Hunting		7	1480	899	2621	661	16	14.1	19.8	1.9	92.2	59.4	151.8	37.1	184.2	117.1	295.7	75.0
Kennel		36	1127	206	3020	538	18.7	3	62.8	10.3	62.4	29.7	99.7	14.8	125.8	54.5	192.9	30.0
Pet		16	1926	544	1878	376	22.8	11.7	31.5	13.1	57.3	41.3	81.7	18.9	124.1	85.9	177.1	38.0
Activity level^2^																		
High		15	1694	899	4014	801	18	14.1	23.3	2.6	93.9	59.4	211.3	42.3	193.2	117.1	441.1	87.2
Moderate		5	1267	715	1695	409	20.6	11.7	31.5	7.5	63.4	52	90.8	15.8	132.7	108.0	184.0	30.3
Low		42	1213	338	2510	495	19.4	5	33.9	11.6	63.3	29.7	110.1	20.2	130.7	54.5	240.5	40.5
Resting		8	1483	206	3020	868	28.3	3	62.8	17.6	54.2	46.5	68.2	7.7	118.1	85.9	146.9	21.8
Method																		
DLW		15	1657	206	4014	1008	19.9	3	62.8	13.1	88.8	46.5	211.3	45.8	180.9	89.9	441.1	95.7
FE		39	1306	376	2178	440	19.4	5	33.9	11.5	68.9	38.2	109.9	20.0	142.2	90.5	229.5	38.9
IC		10	1140	338	1958	615	22.6	10.9	32.0	8.6	47.0	29.7	62.0	11.0	103.1	54.5	146.9	33.1
Tracer		6	1226	544	1695	492	22.4	11.7	31.5	8.9	54.7	46.5	61.1	5.2	117.3	85.9	131.1	16.8

1 Size subjectively scored as toy, small, medium, large, and giant, using the criteria of Hawthorne et al 2004 [18] and Hosgood & Scholl 1998 [85].

2 For most groups, activity level was classed as low (<1 h/day), medium (1–3 h/day), high (>3 h/day) based upon the criteria of Butterwick and Hawthorne (1998) [7]; however, activity levels were classed as ‘resting’ when measurements were taken either when lying still in lateral recumbency or when cage rested. BW: body weight; DLW: doubly-labelled water; F: female; FE: feeding experiment; I: intact; IC: indirect calorimetry; MER: maintenance energy requirement; M: male; Mix: study contains a mix of sexes or different neuter status; N: number of treatments; Neu: neutered; SD: standard deviation.

#### Husbandry and method of determining maintenance energy requirement

In total, 36 and 16 treatment groups comprised kennel dogs and pet dogs, respectively, with the remainder comprising either hunting (7 treatment groups), racing (7 treatment groups), or working (4 treatment groups, all sheepdogs) dogs (Table 3). As indicated in Table 3, the dogs in most treatment groups were classified as having low activity (<1 h/day; 42 treatment groups), with lesser numbers described as having high activity (>3 h/day; 15 treatment groups), moderate activity (1–3 h/day; 5 treatment groups) or resting (8 treatment groups). Maintenance energy requirements were assessed with FE in 39 treatment groups, with IC in 10 treatment groups, and with tracer studies in 21 treatment groups (15 of which were DLW studies) (Table 3).

### Metabolisable energy requirements for maintenance

Overall, mean bodyweight was 20.1±8.8 kg (range 3.0–62.8 kg), whilst maintenance energy requirements were 1351±639.8 kcal/day (range 206–4014 kcal/day), or 142.8±55.3kcal.kgBW^−0.75^.day^−1^ (range 54.5–441.1 kcal.kgBW^−0.75^.day^−1^) (Table 3). Information regarding the effects of activity level, age range, husbandry type, method used to determine energy requirement, neuter status, sex, and dog size on maintenance energy requirements for dogs are reported in Table 4.

**Table 4:** The effects of signalment, husbandry, activity and methodology on maintenance energy requirements (MER) in the adult dog.

		MER (kcal.day^−1^)				MER (kcal.kgBW^−1^.day^−1^)				MER (kcal.kgBW^−0.75^.day^−1^)			
		Mean	SD	CI lower	CI upper	Mean	SD	CI lower	CI upper	Mean	SD	CI lower	CI upper
Size^1^													
	Giant	3477	380.9	2808	4087	69.8	15.8	43.3	96.3	181.8	32.9	126.5	237.1
	Large	1847	163.5	1572	2121	69.7	9.8	53.3	86.1	159	18.7	127.6	190.4
	Medium	1375	184.6	1065	1685	79	11	60.5	97.5	159.5	21.1	124	194.9
	Toy	633	256.1	203	1063	89.9	16.8	61.7	118.2	136.6	30.8	84.6	188
	Not known	1849	258.4	1416	2283	95.6	15.2	70	121.1	198.4	29.3	149.1	247.6
	P-value^2^	<0.001				0.29				0.23			
Age range													
	Old	1836	250.2	1416	2256	84.3	14.6	59.7	108.8	172	28.3	124.4	219.6
	Adult	1849	204.3	1506	2192	81.8	11.9	61.9	101.8	168.7	22.8	130.5	207
	Young adult	1836	186.6	1522	2149	79.7	10.4	62.3	97.1	165.6	20.1	131.8	199.4
	Not given	1801	205.4	1456	2146	77.4	12.2	56.9	97.9	161.6	23.3	122.5	200.7
	P-value^2^	1.00				0.98				0.99			
Sex													
	F	1676	199.9	1340	2011	81.7	11.8	61.9	101.6	163.5	22.6	125.6	201.4
	M	1792	206.4	1626	2318	89.8	11.5	70.5	109.1	184.5	22.4	146.9	222.1
	Mix	1843	200.1	1507	2179	70.9	11.9	50.9	90.8	153	22.7	114.9	191.1
	P-value^2^	0.30				0.26				0.39			
Neuter status													
	I	2090	210.3	1737	2443	94.4	12.2	74	114.8	195.7	23.4	156.4	234.9
	Neu	1724	215.6	1362	2086	69.1	12.6	47.9	90.3	146.4	21.5	105.8	187
	Mix	1678	142.8	1438	1917	78.9	8	65.5	92.3	158.9	15.4	133	184.7
	P-value^2^	<0.001				<0.001				<0.001			
Husbandry													
	Racing	2227	282.5	1753	2700	99.1	15.7	72.7	125.5	202.9	30.6	151.6	254.1
	Working	1978	284	1501	2454	91.2	16.2	64	118.3	188.6	31.5	135.7	241.4
	Hunting	1915	333.8	1355	2476	78	19.6	45.1	110.9	164.8	37.7	101.5	228.1
	Kennel	1399	164.1	1123	1674	68.9	9.5	53	84.7	137.7	18.1	107.3	168.1
	Pet	1833	207.5	1285	1981	66.9	11.7	47.2	86.6	141	22.7	103	179.1
	P-value^2^	<0.001				0.09				0.007			
Activity level^2^													
	High	1946	233.8	1553	2338	93.9	12.8	73.2	115.4	192.6	25.2	150.3	234.9
	Moderate	1833	200.7	1496	2170	81.5	11.5	62.3	100.7	167.4	22.1	130.2	204.6
	Low	1798	197.3	1467	2129	76.2	10.9	57.9	94.5	158.8	21.2	123.2	194.5
	Resting	1744	202	1405	2083	71.7	11.5	52.3	91	149.1	22.3	111.8	186.5
	P-value^2^	0.62				0.07				0.09			
Method													
	DLW	2070	191.1	1750	2391	93.6	11.1	75	112.3	193.5	21.4	157.5	229.4
	FE	1881	198.4	1548	2214	85.9	11.4	66.7	105.1	176.1	22	139.2	231
	IC	1754	230.3	1638	2141	68.4	12.5	47.4	89.4	144.5	24.5	103.4	185.7
	Other tracer	1615	355.8	1051	2179	75.3	20.1	41.5	109.1	153.9	38.3	89.6	218.1
	P-value^2^	0.39				0.19				0.20			

1 Size subjectively scored as toy, small, medium, large, and giant, using the criteria of Hawthorne et al 2004 [18] and Hosgood & Scholl 1998 [85].

2 *P*-values representative the results of comparisons amongst signalments. For most groups, activity level was classed as low (<1 h/day), medium (1–3 h/day), high (>3 h/day) based upon the criteria of Butterwick and Hawthorne (1998) [7]; however, activity levels were classed as ‘resting’ when measurements were taken either when lying still in lateral recumbency or when cage rested. BW: body weight; CI: confidence interval; DLW: doubly-labelled water; F: female; FE: feeding experiment; I: intact; IC: indirect calorimetry; MER: maintenance energy requirement; M: male; Mix: study contains a mix of sexes or different neuter status; Neu: neutered; SD: standard deviation.

#### Effect of breed, age, sex and neuter status

Not surprisingly, when assessed in terms of metabolisable energy per day (kcal.day^−1^), there was a significant breed size effect (*P*<0.001), but the effect disappeared when size was factored in (kcal.kgBW^−1^.day^−1^, *P* = 0.29; kcal.kg.BW^−0.75^.day^−1^, *P* = 0.23; Table 4). The age of the dog had no effect on maintenance energy requirements, with young adult dogs (79.7±10.4 kcal.kgBW^−1^.day^−1^; 165.6±20.1 kcal.kgBW^−0.75^.day^−1^) having similar requirements to old adult dogs (84.3±14.6 kcal.kgBW^−1^.day^−1^; 172±28.3 kcal.kgBW^−0.75^.day^−1^, *P* = 0.99).

There was no effect of sex on maintenance energy requirements of dogs, with similar requirements in male (184.5±22.4 kcal.kgBW^−0.75^.day^−1^) and female (163.5±22.6 kcal.kgBW^−0.75^.day^−1^) dogs (*P* = 0.39). In contrast, the neuter status of the dog did have a significant effect, with the maintenance energy requirements of neutered dogs (146.4±21.5 kcal.kgBW^−0.75^.day^−1^) being less than the requirements of intact dogs (195.7±23.4 kcal.kgBW^−0.75^.day^−1^; *P*<0.001).

#### Effects of husbandry and activity

The husbandry setting of the dog had a significant effect on maintenance energy requirements (kcal.kgBW^−0.75^.day^−1^) (*P* = 0.007; Table 4). Not surprisingly, racing dogs had the greatest energy requirements (202.9±30.6 kcal.kgBW^−0.75^.day^−1^), followed by working (188.6±31.5 kcal.kgBW^−0.75^.day^−1^) and hunting (164.8±37.7kcal.kgBW^−0.75^.day^−1^) dogs. The energy requirements of pet dogs (141.0±22.7 kcal.kgBW^−0.75^.day^−1^) were similar to those of kennel dogs (137.7±18.1 kcal.kgBW^−0.75^.day^−1^). Interestingly, however, a trend was evident whereby maintenance energy requirements were greater in dogs that were more active (Table 4; *P* = 0.09).

#### Methodology effects

The method used to investigate maintenance energy requirements did not have any effect on reported energy requirements (Table 4, *P* = 0.20).

### Generation of allometric equations

The back-transformed allometric equations determined the daily maintenance energy requirement of all study dogs to be 81.5 kcal.kgBW^−0.93^.day^−1^. However, there was large variability, and the model fitted the data only moderately well (Adjusted R^2^ 0.64; Table 5). The effects of signalment factors were then assessed.

**Table 5:** Allometric equations (Y = aBW^b^) for maintenance energy requirements of adult dogs based on the log-log regression of metabolisable energy (kcal.day^−1^) and body weight (kg).

		N	Constant coefficient (a)	SE	Kcal equivalent^1^	Log BW (b)	SE	Adjusted R^2^ (%)	P-value
Overall		70	1.91	0.11	81.5	0.93	0.08	63.5	<0.001
									
Size^2^									
	Giant	1	*	*	*	*	*	*	*
	Large	22	2.99	0.38	986.3	0.17	0.27	0	0.54
	Medium	34	5.15	2.97	141253.8	−1.33	2.54	0	0.6
	Toy	1	*	*	*	*	*	*	*
	Not given	12	2.08	0.23	120.5	0.8	0.18	66.2	<0.001
Age range									
	Old	2	*	*	*	*	*	*	*
	Adult	9	1.9	0.04	79.3	0.88	0.03	99.3	<0.001
	Young adult	33	2.02	0.13	103.8	0.86	0.11	66.5	<0.001
	Age not given	26	1.32	0.41	21.1	1.39	0.32	43.1	<0.001
Sex									
	F	20	1.54	0.38	34.8	1.24	0.34	39.3	<0.001
	M	8	2.35	0.18	225.4	0.59	0.13	75	0.003
	Mix	42	2.03	0.15	107.4	0.85	0.11	59.2	<0.001
Neuter status									
	I	7	2.19	0.39	155.6	0.75	0.3	46.8	0.05
	Neu	2	*	*	*	*	*	*	*
	MIX	61	1.89	0.12	77.8	0.94	0.09	64.2	<0.001
Husbandry									
	Racing	7	4.14	0.45	13899.5	−0.54	0.31	24.3	0.15
	Working	4	2.83	1.87	676.1	0.26	1.49	0	0.88
	Hunting	7	1.52	1.78	33.1	1.35	1.49	0	0.41
	Kennel	36	1.88	0.1	76.4	0.92	0.08	77.5	<0.001
	Pet	16	1.8	0.18	62.5	0.97	0.13	78.6	<0.001
Activity level^3^									
	High	15	1.95	0.88	88.9	0.99	0.71	6.6	0.18
	Moderate	5	2.06	0.42	115.9	0.79	0.32	55.4	0.09
	Low	42	1.81	0.13	64.3	0.99	0.1	69.5	<0.001
	Resting	8	1.84	0.07	69	0.92	0.05	97.9	<0.001
Method									
	DLW	15	2.01	0.25	102.1	0.92	0.2	54.9	<0.001
	FE	39	2.02	0.1	105	0.85	0.08	73.9	<0.001
	IC	10	0.88	0.07	7.6	1.59	0.05	99.2	<0.001
	Other tracer	6	1.71	0.14	50.7	1.02	0.1	95	<0.001

The format of the allometric equations is as follows: Y = aBW^b^; where Y: energy requirement; a: allometric coefficient; BW: bodyweight; b: allometric exponent.

1 Back-transformed using the inverse of log-base10.

2 Size subjectively scored as toy, small, medium, large, and giant, using the criteria of Hawthorne et al 2004 [18] and Hosgood & Scholl 1998 [85].

3 For most groups, activity level was classed as low (<1 h/day), medium (1–3 h/day), high (>3 h/day) based upon the criteria of Butterwick and Hawthorne (1998) [7]; however, activity levels were classed as ‘resting’ when measurements were taken either when lying still in lateral recumbency or when cage rested. DLW: doubly-labelled water; F: female; FE: feeding experiment; I: intact; IC: indirect calorimetry; M: male; Mix: study contains a mix of sexes or different neuter status; N: number of treatment groups; Neu: neutered; SE: standard error.

#### Breed, age, sex, and neuter status effects

As indicated by the poor Adjusted R^2^ values (Table 5), the available data for breed were too variable to enable reliable individual allometric equations to be determined for dogs based on breed size. When effects of age were assessed, adult dogs had a daily maintenance energy requirement of 79.3 kcal.kgBW^−0.88^.day^−1^ (Adjusted R^2^ 0.99). A further equation was generated using data from young adults only (103.8 kcal.kgBW^−0.86^.day^−1^), and this model was a moderate fit (Adjusted R^2^ 0.66). In contrast, equations for old dogs could not be generated because not enough treatment groups were available for analysis. Estimated daily maintenance energy requirements for male and female dogs were 225 kcal.kgBW^−0.59^.day^−1^ and 34.8 kcal.kgBW^−1.24^.day^−1^, but only the model for male dogs was a reasonable fit (Adjusted R^2^ 0.75). Further data for entire and neutered dogs were too variable for the models generated to be acceptable (Table 5).

#### Husbandry and activity effects

Pet dogs had a mean daily energy requirement of 62.5 kcal.kgBW^−0.97^.day^−1^ (Adjusted R^2^ 0.79), whilst kennel dogs had a mean requirement of 76.4 kcal.kgBW^−0.92^.day^−1^ (Adjusted R^2^ 0.78). Unfortunately, either high variability or low numbers of treatment groups in each category meant that the effects of husbandry on daily maintenance energy requirements could not be determined for hunting, racing and working dogs (Table 5).

Estimates of daily maintenance energy requirements were most reliable for dogs classed as resting (69 kcal.kgBW^−0.92^.day^−1^; Adjusted R^2^ 0.98) or with low activity levels (64.3 kcal.kgBW^−0.99^.day^−1^; Adjusted R^2^ 0.70; Table 5). In contrast, there was more variation in the data for dogs classed as having moderate and high activity levels, meaning that reliable estimates could not be made (moderate activity Adjusted R^2^ 0.55; high activity, Adjusted R^2^ 0.07).

#### Methodology effects

Different allometric equations were generated for the different methods used to determine energy requirements (Table 5). Equations determined from studies using IC (7.6 kcal.kgBW^−1.59^.day^−1^, adjusted R^2^ 0.99) and tracer studies (50.7 kcal.kgBW^−1.02^.day^−1^; adjusted R^2^ 0.95) were most reliable, with equations derived from feeding studies (105 kcal.kgBW^−0.85^.day^−1^; adjusted R^2^ 0.75) and those using DLW (102 kcal.kgBW^−0.92^.day^−1^; adjusted R^2^ 0.55) less reliable (Table 5).

### Energy requirements in pet dogs

The maintenance energy requirements of pet dogs were investigated in a total of 16 treatment groups, the majority of which were of either medium (7 treatment groups) or large (5 treatment groups) breed (Table 6). Unfortunately, age was not reported in the majority of pet dogs studied (10 treatment groups; Table 6); where age was reported, most were either classed as adult (4 treatment groups) or young adult (2 treatment groups). The majority (11 treatment groups) of pet dogs were classed as mixed sex, with 5 treatment groups having all male dogs, whilst all subjects were of mixed neuter status (i.e., a mix of neutered and entire dogs). Most pet dogs had low activity (8 treatment groups), followed by moderate activity (4 treatment groups) and resting categories (3 treatment groups). Only one treatment group was classed as high activity. Tracer studies were used in 6 treatment groups, followed by feeding studies (5 treatment groups) and indirect calorimetry (4 treatment groups; Table 6).

**Table 6:** Maintenance energy requirements (MER) and bodyweights for the pet dogs in the final dataset.

			MER (kcal.day^−1^)				BW				MER (kcal.kgBW^−1^.day^−1^)				MER (kcal.kgBW^−0.75^.day^−1^)			
		N treatments	Mean	Min	Max	SD	Mean	Min	Max	SD	Mean	Min	Max	SD	Mean	Min	Max	SD
Overall		16	1296	543.6	1878	370.1	22.8	11.7	31.5	6.2	57.3	41.3	81.7	10.5	124.1	85.9	177.1	22.7
																		
Size^1^																		
	Large	4	1524	1285	1695	205.1	27.8	24	31.5	4.3	55.1	53.6	59.3	2.8	126.2	118.5	131.1	5.4
	Medium	7	1096	543.6	1878	445.1	17.2	11.7	23.3	4.2	63.4	46.5	81.7	13.7	127.1	85.9	177.1	32.8
	Not known	5	1394	1069	1610	224.6	26.8	24	28	1.8	51.9	41.3	57.5	6.2	118.1	93.8	132.3	15.2
Age range																		
	Adult	4	1524	1285	1695	205.1	27.8	24	31.5	4.3	55.1	53.6	59.2	2.8	126.2	118.5	131.1	5.4
	Young adult	2	629.4	543.6	715.2	121.3	11.7	11.7	11.7	0	53.8	46.7	61.1	10.4	99.5	85.9	113	19.2
	Not given	10	1338	971.5	1878	297.8	23.1	17.5	28	4.4	58.8	41.3	81.7	12.7	128.1	93.1	177.1	25.6
Sex																		
	F	2	629.4	543.6	715.2	121.3	11.7	11.7	11.7	0	53.8	46.5	61.1	10.4	99.5	85.9	113	19.2
	M	5	1505	1285	1695	183	25.7	17.5	31.5	5.9	60.4	53.6	81.7	12.1	134.3	118.5	167	18.8
	Mix	9	1329	971.5	1878	314.2	23.7	18	28	4.2	56.3	41.3	80.6	10.4	123.8	93.1	177.1	22.9
Activity level^2^																		
	High	1	1878				23.3				80.6				177.1	*	*	*
	Moderate	4	1201	715.2	1695	440.4	21.5	11.7	31.5	8.4	56.5	51.9	61.1	4.3	119.9	108	131.1	11.1
	Low	8	1317	1048	1610	229.4	23.6	17.5	28	4.7	57	41.3	81.7	11.3	124.5	90.1	167	20.6
	Resting	3	1175	543.6	1695	583.8	22.4	11.7	31.5	10	51.3	46.5	53.8	4.2	110.7	85.9	127.5	21.9
Method																		
	DLW	1	1426	*	*	*	17.5				81.7				167	*	*	*
	FE	5	1249	971.5	1878	367.2	20.6	18	24	2.8	59.9	51.9	80.6	11.9	127.9	108	177.1	28.9
	IC	4	1428	1069	1610	243.9	27.5	25.9	28	1.1	51.8	41.3	57.5	7.2	118.7	93.1	132.3	17.4
	Other tracer	6	1226	543.6	1695	491.8	22.4	11.7	31.5	8.9	54.7	46.5	61.1	5.2	117.3	85.9	131.1	16.8

1 Size subjectively scored as toy, small, medium, large, and giant, using the criteria of Hawthorne et al 2004 [18] and Hosgood & Scholl 1998 [85].

2 For most groups, activity level was classed as low (<1 h/day), medium (1–3 h/day), high (>3 h/day) based upon the criteria of Butterwick and Hawthorne (1998) [7]; however, activity levels were classed as ‘resting’ when measurements were taken either when lying still in lateral recumbency or when cage rested. BW: body weight; DLW: doubly-labelled water; F: female; FE: feeding experiment; IC: indirect calorimetry; MER: maintenance energy requirement; M: male; Mix: study contains a mix of sexes; SD: standard deviation.

Within the pet dog subcategory, age range, method, sex and breed did not affect maintenance energy requirements (Table 7), but there was an effect of activity level (*P*<0.001). Unsurprisingly, pet dogs with high activity levels had the greatest maintenance energy requirements (183.1±23.4 kcal.kgBW^−0.75^.day^−1^), and resting dogs had the least (95.7±11.7 kcal.kgBW^−0.75^.day^−1^). Surprisingly, pet dogs classed as having moderate activity levels (1–3 h/day; 114.1 kcal.kgBW^−0.75^.day^−1^) had lower maintenance energy requirements than those classed as having low activity levels (1 h/day; 125.4 kcal.kg BW^−0.75^.day^−1^).

**Table 7:** The effects of signalment factors on maintenance energy requirements (MER) in the pet dog.

	MER (kcal.day^−1^)				MER (kcal.kgBW^−1^.day^−1^)				MER (kcal.kgBW^−0.75^.day^−1^)			
	Mean	SD	CI lower	CI upper	Mean	SD	CI lower	CI upper	Mean	SD	CI lower	CI upper
Size^1^												
Large	1361	422.5	509.4	2212	50.8	11.3	27.9	73.6	114.9	28.4	57.6	172.2
Medium	1244	200	841	1647	67.4	5.3	56.8	78	138.6	12.7	113.1	164.1
Not known	1437	419.3	592.4	2282	62.3	10.8	40.6	83.9	135.2	24.6	85.8	184.7
P-value	0.84				0.3				0.7			
Age range												
Adult	1347	155.1	1035	1660	60.2	4.3	51.5	68.9	129.6	11.6	106.2	152.9
Young adult	1347	155.1	1035	1660	60.2	4.3	51.5	68.9	129.6	11.6	106.2	152.9
Not given	1347	155.1	1035	1660	60.2	4.3	51.5	68.9	129.6	11.6	106.2	152.9
P-value	0.99				0.99				0.99			
Sex												
F	1001	319.9	356.3	1645	56	8.9	38.1	73.9	112.5	24	64.2	160.8
M	1701	304	1088.4	2314	74.3	7.8	58.5	90.1	161.9	18	125.7	198.2
Mix	1340	190.4	956.3	1724	50.2	5.3	39.5	60.8	114.3	14.1	85.9	142.8
P-value	0.35				0.05				0.14			
Activity level^2^												
High	2020	572.9	856.9	3175	82.9	14.1	54.4	111.3	183.1	23.4	125.9	240.3
Moderate	1114	146	819.4	1408	54.2	4.1	45.9	62.5	114.1	11.3	91.3	136.9
Low	1207	267.3	668.3	1745	59.8	6.8	46	73.6	125.4	15.5	94.3	156.6
Resting	1048	159.1	727.7	1369	43.8	4.4	34.9	52.6	95.7	11.7	72.1	119.3
P-value	<0.001				<0.001				<0.001			
Method												
DLW	1316	188.5	936	1696	60.6	5.1	50.3	70.9	129.8	13.2	103.2	156.3
FE	1316	188.5	936	1696	60.6	5.1	50.3	70.9	129.8	13.2	103.2	156.3
IC	1442	342.4	751.9	2132	58.7	9.4	40.5	76.8	129	21.7	85.2	172.7
Other tracer	1316	188.5	936	1696	60.6	5.1	50.3	70.9	129.8	13.2	103.2	156.3
P-value	0.76				0.85				0.97			

1 Size subjectively scored as toy, small, medium, large, and giant, using the criteria of Hawthorne et al 2004 [18] and Hosgood & Scholl 1998 [85].

2 For most groups, activity level was classed as low (<1 h/day), medium (1–3 h/day), high (>3 h/day) based upon the criteria of Butterwick and Hawthorne (1998) [7]; however, activity levels were classed as ‘resting’ when measurements were taken either when lying still in lateral recumbency or when cage rested. BW: body weight; CI: confidence interval; DLW: doubly-labelled water; F: female; FE: feeding experiment; IC: indirect calorimetry; MER: maintenance energy requirement; M: male; Mix: study contains a mix of sexes; SD: standard deviation.

**Figure 2:**
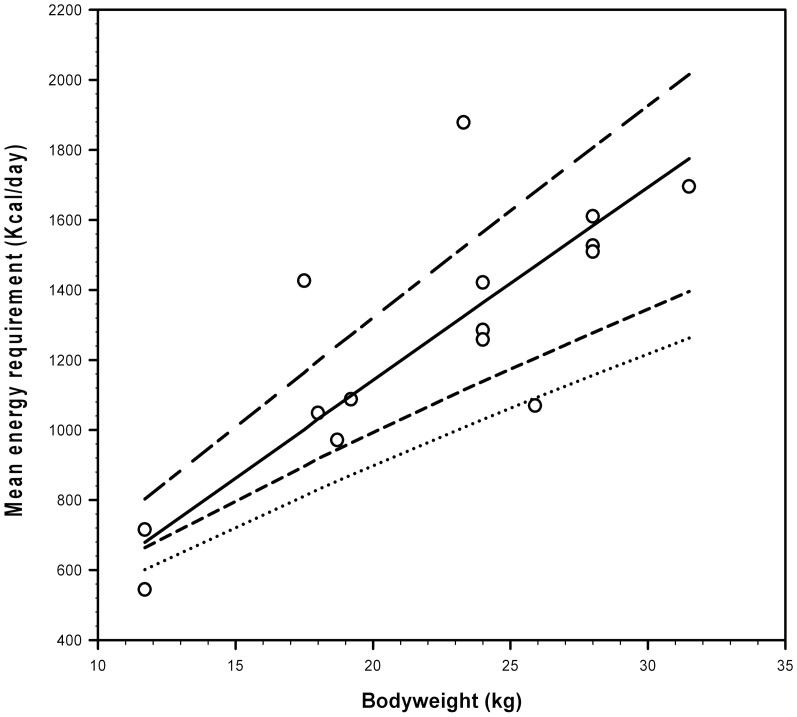
Effect of bodyweight (BW) on the maintenance energy requirements (MER; kcal/d) in the pet dog (open circles), compared with the predicted requirements from the present study for all dogs (line with alternating long and short dashes; 81.5 kcal.kgBW^−0.93^.day^−1^) and pet dogs only (solid line; 62.5 kcal.kgBW^−0.97^.day^−1^). For comparative purposes, lines are also included depicting NRC 2006 [1] estimates for inactive (dotted line; 95.0 kcal.kgBW^−0.75^.day^−1^) and active line with short dashes; 105.0 kcal.kgBW^−0.75^.day^−1^) pet dogs.

The ability of allometric equations to predict the maintenance energy requirements of pet dogs from the current study (62.5 kcal.kgBW^−0.93^.day^−1^) were compared to those predicted for inactive pet dogs (95.0 kcal.kgBW^−0.75^.day^−1^) and active pet dogs (105.0 kcal.kgBW^−0.75^.day^−1^) by the NRC [1] (Figure 2). The allometric equation from the current study provided the best fit for the data, being superior to the NRC equations, both of which underestimated requirements, especially at heavier bodyweights.

## Discussion

This meta-analysis aimed to determine the maintenance energy requirement of adult dogs using available published information. Although an evidence-based review has previously been undertaken examining nutritional and other management of obesity [29], to the authors' knowledge, this is the first meta-analysis to be conducted in the field of canine clinical nutrition. Meta-analyses are a subcategory of systematic review and, as such, represent a superior level of evidence to individual trials [30], given their ability to examine large datasets and to minimise bias from individual publications. In this study, the final data set was obtained from 29 independent publications, comprising 70 separate treatment groups of adult dogs, covering a wide spectrum of breeds, body size, sexual status, age, husbandry conditions, and activity levels. The information it provides should be of use to veterinarians, nutritionists, pet food manufacturers, and pet owners, in understanding the nutritional requirements of dogs.

Average maintenance energy requirements of adult dogs were 142.8±55.3 kcal.kgBW^−0.75^.day^−1^, corresponding to an allometric equation of 81.5 kcal.kgBW^−0.93^.day^−1^. A number of factors affected maintenance energy requirements including husbandry, and neuter status. A trend also existed for an effect of activity level on energy requirements of all dogs. The majority of dogs studied were maintained in a kennel environment, including those from laboratory settings, with a proportionately lesser contribution from pet dogs, working dogs and hunting dogs. As previously suggested [31], care must be taken when extrapolating such data since they may not be representative of dogs maintained in different husbandry settings, including pet dogs in the home. For this reason, data from pet dog studies were examined separately, and average maintenance energy requirements for this subgroup were less than for the full dataset (124.1±38.0 kcal.kgBW^−0.75^.day^−1^), with a predicted allometric equation of 62.5 kcal.kgBW^−0.97^.day^−1^. In this subgroup, the level of activity was the main factor of significance, with maintenance energy requirements, being greatest in the most active dogs, and least in resting dogs. This confirms previous recommendations that adult pet dogs have different maintenance energy requirements from other populations.

Given current concerns regarding the prevalence of obesity in companion animals [32], this review placed specific emphasis on determining maintenance energy requirements for pet dogs. Reliable data from this cohort were limited, with only 16 treatment groups (23% of the groups included) meeting eligibility requirements for inclusion in the final meta-analysis. The main reasons for exclusion were the fact that the data were presented in a format which precluded their use (i.e., only presented in graphical form [7], [8], or because the energy requirements were estimated from client survey data [5], [6]. We chose to exclude survey data for the current review due to the inherent difficulties in obtaining accurate information. Under-reporting is a well-known phenomenon in human nutritional studies [13], [14], [15], [33], making the accuracy of data obtained in this manner highly questionable. Indeed, for this reason, the UK's Scientific Advisory Committee on Nutrition, chose to base the latest dietary reference values for human energy requirements on DLW measurements, and to disregard studies using self-reported food intake [34]. Owner estimates of food intake in pet dogs are thought to be similarly flawed [35], with errors in owner recall of food information, errors in the measurement of food portions [36], regular switching between different foods, the use of home-cooked recipes, and feeding treats and table scraps [37]. Finally, the method by which energy content of food is determined might be important, namely whether measured in feeding trials, or calculated and, if so, by what method (modified Atwater factors [38] or NRC 2006 [1]). Calculations based upon modified Atwater factors often under-estimate the actual energy content of commercial foods of dogs [35]. Therefore, for studies based upon survey data, actual energy intake could be under-estimated if the energy content of a significant number of diets were estimated in this way.

The discrepancy between maintenance energy requirements determined from feeding survey data and data derived from other methods is illustrated by the fact the most recent study using feeding survey data suggested requirements of 97.8 kcal.kgBW^−0.75^.day^−1^ [5], markedly less than the estimate for pet dogs from the current study (124.1 kcal.kgBW^−0.75^.day^−1^). Future studies on pet dogs should consider using more robust methods of determining energy requirements, which could include using food laboratories [39], [40], or methods such as DLW measurement, the preferred method to determine energy requirements in human studies of [34]. In addition, more objective measures of physical activity could be considered. In humans, Techniques such as heart rate monitoring (HRM) and accelerometry provide minute-by-minute data and give information on the total levels of physical activity, as well as its intensity, duration and frequency [34]. Indeed, accelerometers have been validated for use in dogs [41].

The allometric equation generated for pet dogs in the current study (e.g. 62.5 kcal.kgBW^−0.93^.day^−1^) was a more accurate predictor of average maintenance energy requirements than the equations currently recommended by the NRC [1] (Figure 2). This equation was generated from the data from pet dogs only, and the better accuracy may partly be explained by the use of stringent eligibility criteria, which excluded data derived from less reliable methods of measurement. However, despite an improved ability to predict the mean, the marked variability in requirements within the population must be emphasised and, arguably, as with previous equations, the mean requirement will not adequately reflect the actual requirement for many dogs. Similar variability is seen with energy requirements in man [34]. Such variability may be explained by the fact that energy requirements comprise a number of components including the basal metabolic rate, diet-induced thermogenesis, energy required for physical activity, and thermoregulation [1]. Diet-induced thermogenesis can vary as a result of meal frequency [42], and dietary macronutrient content especially protein, although the contribution from the latter is relatively minor [1]. These factors were not specifically assessed in the meta-analysis, and could account for some of the unexplained variability. Furthermore, factors such as environment and seasonality were also not assessed, and could well have contributed further to the variability in the estimates of energy requirement.

Whatever the reason for the variability in maintenance energy requirements estimates, caution is recommended when using this, or any other, allometric equation to predict maintenance energy requirements, and it is critical to adjust intake based upon response. Follow-up, based upon weight and BCS measurements, is simple and non-invasive, although it requires a change in mind-set of practising veterinarians. In this respect, first opinion practitioners do not regularly perform body condition scores, and body weight is typically measured only once every four consultations [43]. A further concern with recommendations for maintenance energy requirements is the fact that common methods for measuring food portions tend to over-estimate portion size [36], and many pet dogs receive considerable additional food from treats and table scraps [37]. To ensure feeding of appropriate amounts, owners should be counselled about using accurate methods to determine portion sizes such as with electronic scales. Furthermore, owners should be made aware of the dangers of feeding additional food, particularly table scraps, and of the need to take such additional food into account when determining the daily food intake of an individual dog.

Perhaps surprisingly, there was not a consistent effect of activity on maintenance energy requirements in dogs. This may be due to limitations in the methodology used to quantify activity, and diversity in the types of dog represented including racing, hunting, working, and pet dogs. In the current study, we classified activity based upon the time spent exercising [17], but this classification did not take account intensity of activity, which is likely to have a marked effect on energy consumption. For example, both greyhounds and huskies would be classed as racing dogs, but the nature of the exercise differs greatly between them: greyhounds typically undertake short bouts of extreme activity, often covering a distance of 500 m in a 33s race [44]. In contrast, huskies undertake long periods of endurance activity. During races such as the Iditarod trail, the dogs travel 700 km over a period of 10 days [45]. Not surprisingly, therefore, estimates of maintenance energy requirement were least reliable (i.e. R^2^ was worst) for dogs with moderate and high activity levels. For future studies involving active dogs, the energy cost of different types of exercise should be better defined, since this would permit maintenance energy requirements to be more accurately defined in different groups of racing dog. Further, objective methods of measuring physical activity should be used in preference to subjective methods, such as HRM and accelerometry, as recommended for humans [34]. The main advantage of such an approach is that it is then possible to tailor dietary reference values for energy values based upon the amount of physical activity undertaken [34].

In contrast to the complete dataset, activity level was found to be of importance in influencing energy requirements of the pet dogs in the study. Not surprisingly, maintenance energy requirements for those dogs undertaking high levels of activity were greater than for other groups. However, maintenance energy requirements for dogs classed as having moderate activity (114.1 kcal.kgBW^−0.75^.day^−1^) were not significantly different from dogs classed as resting (95.7 kcal.kgBW^−0.75^.day^−1^), and less than dogs reported to have low activity levels (125.4 kcal.kgBW^−0.75^.day^−1^). This discrepancy both questions the reliability of reporting of activity levels in the publications, which were often based upon owner reports, and also suggests that the typical exercise that most pets undertake is minimal and does not markedly alter energy requirements. Most pet dogs are inactive or only moderately active [17], with median weekly activity equating to only 4 walks of 40 minutes each, and 40% of pet dogs not being walked at all [46]. These low activity levels are in contrast to owner perceptions, with most believing that their dogs receive adequate exercise, even though some receive no exercise at all [47].

Therefore, although our findings indicate that activity, most notably high activity levels, influence the maintenance energy requirements in pet dogs, the challenge for the veterinary profession is to develop clear guidelines that will not be misinterpreted by dog owners. Currently, many commercial foods provide different recommendations for active and inactive pet dogs. However, given the tendency for owners to overestimate the activity of their dog reliably, it would be preferable either not to include different recommendations based upon activity, or to choose the terminology used carefully. For instance, rather than using terms such as “inactive” or “low activity”, the term “typical pet” or “standard” might be more appropriate. Further, it might be preferable to use the terms “very active” or “working dog” to note an energy requirement for the minority of dogs with genuinely increased activity levels.

The majority of publications examined used dogs of both sexes, or did not specify the sex, making it difficult to determine the effects of sex and neuter status on maintenance energy requirements. In kennelled dogs, maintenance energy requirements were greater in entire than in neutered females, but this difference was not evident in either working dogs or pet dogs, most likely because of smaller numbers of neutered working and pet dogs in these categories. Although the effects of neutering have been widely investigated in cats [11], work has been less extensive in dogs. Anantharaman-Barr [48] found that energy expenditure decreased by 30 days after neutering in mixed-breed female dogs. However, the difference was no longer evident at day 90 post-neutering, probably due to the fact that body weight increased by 7% over this period. Decreases in energy expenditure in neutered animals were also seen in another study [21] and, together with the increase in voluntary food intake that is also observed, suggests that close monitoring is required after neutering to prevent unwanted weight gain.

This meta-analysis included data from studies using a range of experimental methods including DLW, IC, and FE. With FE, direct estimates of the metabolisable energy required for maintenance can be made, provided that body weight remains stable during the experimental period. In contrast, methods such as DLW and IC measure energy expenditure rather than maintenance energy requirements. For this meta-analysis, it was assumed that the dogs in these studies were in energy balance and, therefore, that energy expenditure was equivalent to energy requirement. This is a limitation of the current study, since this might not have been the case. Nonetheless, the same approach has been used when setting energy reference values for humans [34]. The influence of experimental method on maintenance energy requirements was also examined statistically in the current meta-analysis, and there were no significant differences amongst methods. That said, markedly different allometric equations were generated, with constant coefficients varying between 7.6 (IC) and 105 (FE), and exponents varying between 0.85 (FE) and 1.59 (IC). Therefore, whatever the reason for these differences, we would advise that direct comparisons amongst studies using different methodologies should be made cautiously in the future. As well as differences in the allometric equations themselves, marked discrepancies were seen in the reliability of the different allometric equations, with r^2^ varying between 0.55 (DLW) and 0.99 (IC). The reason for this is also not clear, but it is noteworthy that, when allometric equations were generated for dogs with different activity levels, the most reliable equation was generated for resting dogs. Thus, the superior reliability of the allometric equation generated from IC studies might actually be because dogs undergoing IC must be resting during the procedure.

The species *Canis familiaris* is unusual in that it encompasses many different breeds, which vary greatly in size, from toy (e.g., Chihuahua, Papillon) to giant (e.g., Saint Bernard or Great Dane). Breed differences not only have a marked effect on stature and body shape, but also on lifespan and internal anatomy (e.g., the relative size of the digestive tract) [20]. These differences also make it inherently difficult to determine maintenance energy requirements accurately across the species [20], and might explain the fact that no breed size differences were observed in the current study. Typically, the data available were too variable to determine allometric equations reliably for dogs based on breed size, as indicated by poor Adjusted R^2^ values (Table 5). This is likely due to the fact that breed size in the current study was mainly based on BW, meaning that breeds of broadly similar size were grouped. For example, the Husky and Greyhound are both considered to be large dogs, but their shape, body composition, overall volume, and coat characteristics differ, all of which are likely to affect maintenance energy requirements [49], [50]. One limitation of the current study was that data for toy breeds and giant breeds were sparse. Thus, whilst the maintenance energy requirements are likely to be accurate for mid-size and large-breed dogs, caution should be exercised when extrapolating these results to the extremes. The popularity of miniature dog breeds is increasing, relative to other breeds [51], likely due to their convenience and reduced costs. As a result, more data regarding the nutritional requirements of such breeds are needed in the future, to ensure that feeding recommendations are soundly based.

Related to breed differences is the possible effect of age on maintenance energy requirement, since growth, ageing and lifespan differ markedly amongst breeds [20]. Giant dogs are growing until 2 years of age, but their lifespan is considerably shorter than for other breeds such as toy breeds. Thus, biological age depends not only on chronological age, but also upon the breed, and makes interpretation of the effects of age on maintenance energy requirements complicated [52]. Age is likely to have been an additional confounding factor when examining the effect of differences in breed.

Longevity is increasing in companion animals [53] and, with this, comes an increased likelihood of developing chronic diseases such as osteoarthritis and chronic kidney diseases. Therefore, knowledge of the nutrient requirements of ageing pets is an area of increasing importance. In cats, data on the energy requirements of older cats are contradictory, with some publications reporting a decrease in daily maintenance energy requirements at approximately 6–7 years of age [54], [55], whilst others report no affect of age [11], [56], [57]. Ageing in cats may also result in decreased digestibility of nutrients, most notably fat [53], [58], [59]. To the authors' knowledge, few publications have examined the effects of ageing on the physiology of domestic dogs. The limited work conducted to date has suggested that there are no effects on intestinal permeability [60], but changes in intestinal morphology are seen [61]. Thus, more ageing-related work is required in dogs. Unfortunately, allometric equations could not be generated for old dogs because not enough of the treatment groups used in the analysis contained such dogs. This likely reflects the difficulty in obtaining the participation of older dogs in research studies; sporting, hunting, working, and laboratory dogs are often retired before they reach old age, whilst the development of ageing-related diseases can preclude the participation of older pet dogs in research studies. Therefore, older dogs would be a priority for future studies assessing maintenance energy requirements in pet dogs, so that the knowledge base in this area can be improved.

One final limitation of the current study was the fact that data were not available for lean mass or body composition. This is not surprising because determining lean body mass is expensive, invasive, and may not always be practicable, not least for pet dogs. Lean body mass is known to be a better predictor of resting energy expenditure in humans [62], and the best predictor of maintenance energy requirements in cats [11]. As a result, acquiring body composition data should be a priority in future studies of maintenance energy requirements in dogs.

In conclusion, the current meta-analysis has estimated maintenance energy requirements of adult dogs. Although the allometric equation generated was a better estimate for maintenance energy in dogs than previous estimates, great variability in requirements was still seen. Such variability could be reduced if energy requirements were not solely based upon BW data, but included information on the activity level (predominantly for pet dogs), husbandry, and neuter status. For future studies, consideration should be given to generating reference data for energy requirements using objective measurements, such as DLW, in a ‘representative’ target population, and utilising objective measures of determining physical activity such as accelerometry. More attention should also be paid to generating data on the nutritional requirements of older dogs and dogs at the extremes of the body size continuum (i.e., giant and toy breeds). Finally, care should be taken when using the current study findings to develop recommendations for specific groups of dog, most notably those in the pet population. A programme of owner education will be necessary to ensure that overfeeding is avoided, and that caution is exercised when modifying food intake based upon activity levels, given owners' misperception between perceived and actual activity.

## Supporting Information

Spreadsheet S1 Electronic spreadsheet containing the study data used in the current meta-analysis.
(XLSX)

Checklist S1 **Prisma Checklist.**
(PDF)
